# Disparities in clinical studies of AI enabled applications from a global perspective

**DOI:** 10.1038/s41746-024-01212-7

**Published:** 2024-08-10

**Authors:** Rui Yang, Sabarinath Vinod Nair, Yuhe Ke, Danny D’Agostino, Mingxuan Liu, Yilin Ning, Nan Liu

**Affiliations:** 1 Centre for Quantitative Medicine, Duke-NUS Medical School, Singapore, Singapore; 2 Department of Anesthesiology, Singapore General Hospital, Singapore, Singapore; 3 Programme in Health Services and Systems Research, Duke-NUS Medical School, Singapore, Singapore; 4 NUS Artificial Intelligence Institute, National University of Singapore, Singapore, Singapore

**Keywords:** Health care, Medical research

## Abstract

Artificial intelligence (AI) has been extensively researched in medicine, but its practical application remains limited. Meanwhile, there are various disparities in existing AI-enabled clinical studies, which pose a challenge to global health equity. In this study, we conducted an in-depth analysis of the geo-economic distribution of 159 AI-enabled clinical studies, as well as the gender disparities among these studies. We aim to reveal these disparities from a global literature perspective, thus highlighting the need for equitable access to medical AI technologies.

In the rapidly developing field of healthcare, artificial intelligence (AI) has emerged as a pivotal force driving innovation in clinical research and improving the efficiency of clinical studies^1–6^. However, despite rapid technological advancements, its practical application in clinical settings remains limited. Concurrently, there are complex disparities in current AI-enabled clinical studies. These disparities, which include data and algorithms, participants and subjects, and access to cutting-edge technologies^7–10^, challenge the equitable implementation of AI solutions^11,12^.

We identified 159 clinical studies of AI-enabled applications from Embase, MEDLINE, and CINAHL through a systematic review. Among these studies, 109 were conducted in hospital settings, while 50 took place in non-hospital environments. Notably, 51.6% (82/159) of the studies utilized AI for treatment and management, and 40.9% (65/159) focused on AI-assisted diagnosis, with a significant portion related to gastroenterology (see Supplementary Table 1). Moreover, 5.0% (8/159) of the studies applied AI for prognosis, and 2.5% (4/159) studies explored its use in patient education. In this study, we primarily analyzed the geo-economic distributions as well as the gender disparities among the study subjects.

As depicted in Fig. 1a, the majority of studies were conducted in North America, Europe, and East Asia, with the United States (44 studies) and China (43 studies) leading. A significant portion (74.0%) of the clinical studies were implemented in high-income countries, 23.7% in upper-middle-income countries, 1.7% in lower-middle-income countries, and only one clinical study was conducted in low-income countries (i.e., Mozambique), as shown in Fig. 1b. Meanwhile, we analyzed the geo-economic distribution of 318 first and last authors, which revealed a similar distribution pattern. Additionally, we observed that funding status is correlated with country income level; the funding rate in high-income countries was as high as 83.8%, while it was only 68.3% in upper-middle-income countries, as shown in Fig. 1c.

**Fig. 1:**
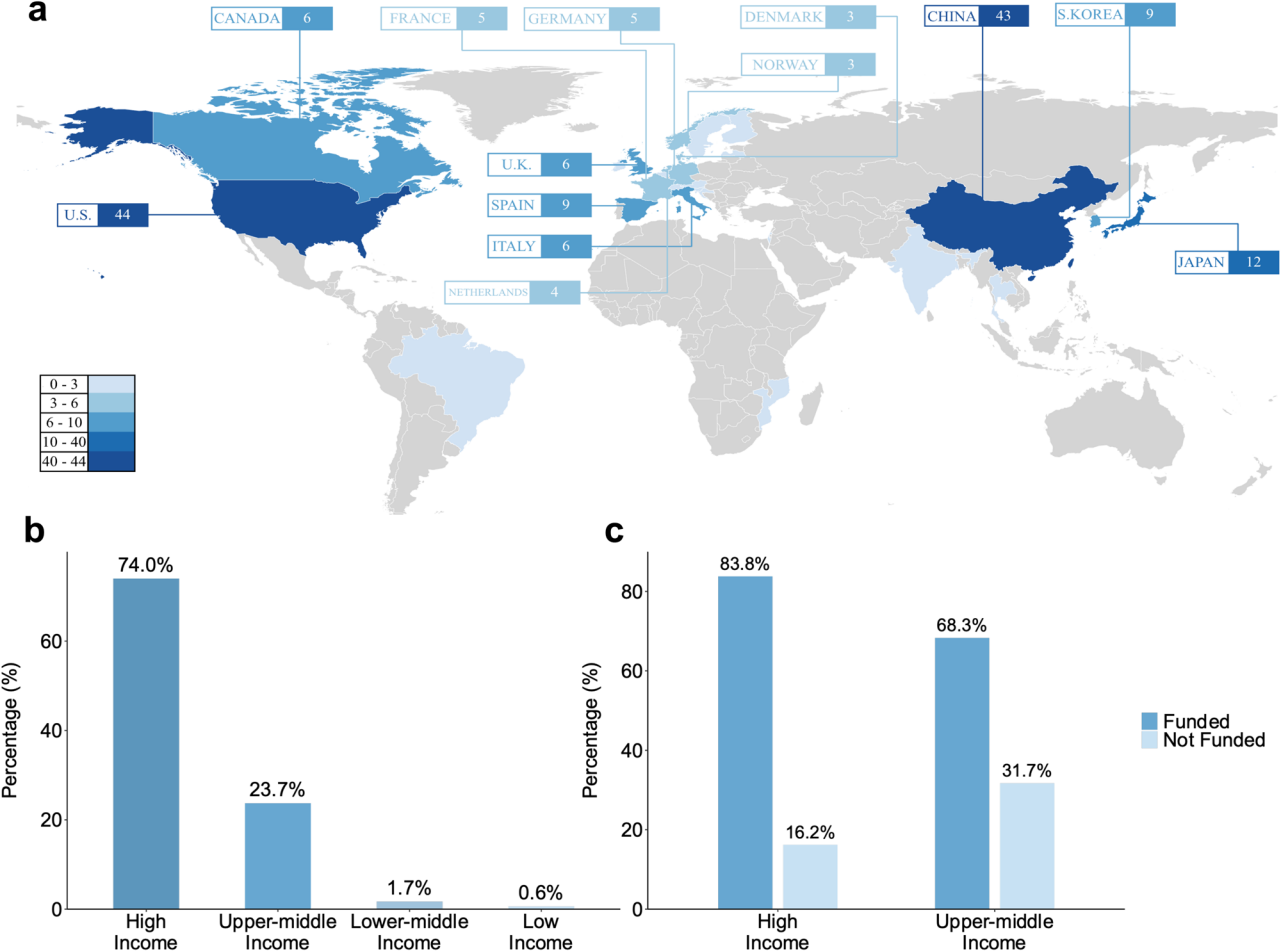
Global view of AI-enabled clinical studies. **a** Geographical distribution of studies. In the figure, we only marked countries that conducted more than two studies. **b** Income level classification of countries conducting studies. **c** Funding status of studies by country income level classification (Only studies conducted in a single country were counted. The fund status of studies in lower-middle-income and lower-income countries is not shown in the figure because the number is only 3, and the bars generated are hardly visible). The map was generated using ArcGIS software.

We further explored the gender disparities among the subjects in these AI-enabled clinical studies. After excluding studies of gender-specific diseases, 146 studies reported gender information, of which only 3 (2.1%) reported an equal number of male and female subjects. We then classified the gender ratio of the remaining studies into three categories: “low disparity (0.7–1)”, “moderate disparity (0.3–0.7]”, and “high disparity (0–0.3]”. As illustrated in Fig. 2, 10.3% (15/146) of the clinical studies exhibited high gender disparity, while another 36.3% (53/146) demonstrated moderate gender disparity. Among the 15 clinical studies with high gender disparity, males predominated in 8 studies, 25% (2/8) of which were related to obstructive sleep apnea (OSA) due to the higher prevalence of OSA in males^13^. Females were the majority in 7 studies, of which 28.6% (2/7) were linked to obesity^14^. Additionally, one study had a higher proportion of female subjects due to the inclusion of a subset of patients undergoing gynecological surgeries. More information is detailed in Supplementary Table 2.

**Fig. 2:**
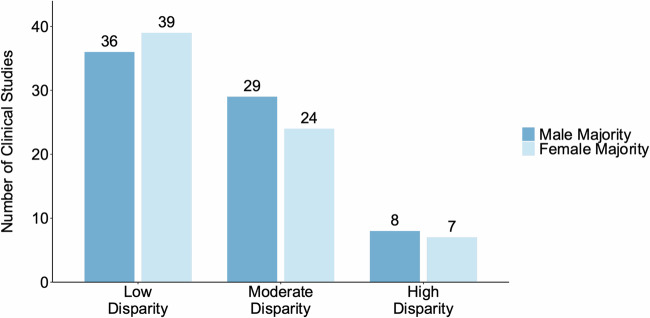
Gender disparities in studies. We categorized the studies into “male majority” and “female majority” groups, depending on whether there were more male or female subjects. Within each group, we calculated the “gender ratio” by dividing the number of subjects from the minority gender by the number from the majority gender. The gender ratio was then classified into three levels based on the gender ratio: “low disparity (0.7–1)”, “moderate disparity (0.3–0.7]”, and “high disparity (0–0.3]”.

In summary, our study examined 159 AI-enabled clinical studies (of which only 6.3% (10/159) were international multicenter studies), and the results indicate significant disparities in both the geo-economic distribution of these clinical studies and the gender of the study subjects.

The leading countries and authors are primarily from high-income and upper-middle-income countries. The limited representation of lower-middle-income and low-income countries is concerning since these countries may have different disease profiles and are, therefore, unable to benefit from existing research^15^. Moreover, the presence of gender disparities in clinical studies could lead to potential health inequalities.

Considering these disparities, it is crucial to involve a diverse group of researchers and gender-balanced subjects in the design of AI-enabled clinical studies. Additionally, international multi-center clinical studies should be encouraged, particularly including sites from lower-middle-income and low-income countries. This will enable AI tools to be effectively applied and evaluated in diverse healthcare systems around the world. The pursuit of health equity will lay a solid foundation for the sustainable development and application of AI in healthcare.

## Methods

### Search strategy

We conducted a systematic literature search using Embase, MEDLINE, and CINAHL to identify AI-enabled clinical studies published prior to January 3, 2024. The search strategy we used included keywords and Medical Subject Headings (MeSH) terms related to “Artificial Intelligence” and “Clinical Study”. Additionally, we manually reviewed the references of the included studies to identify more relevant studies (Detailed search strategy can be found in Supplementary Material Search Strategy and PRISMA flow diagram can be found in Supplementary Fig. 1).

### Inclusion and exclusion criteria

Each article was independently screened by two researchers (R.Y. and S.V.N.) to determine eligibility. Disagreements were resolved through consultation with a third researcher (Y.K.). Studies were included if they: (1) incorporated a significant AI component, defined as a nonlinear computational model (including, but not limited to, support vector machine, decision trees, neural networks, etc.)^6^; (2) were applied in clinical settings, influencing the patient’s health management; and (3) were published as a full-text article in a peer-reviewed, English-language journal. Additionally, we excluded studies that used linear models (such as linear regression and logistic regression), conducted secondary analyses or did not integrate AI algorithms into clinical practice. For each included study, we extracted information including gender information of the subjects, the country of clinical study implementation, the income level of the country (based on World Bank’s classifications^16^), the funding, and so on. All extracted information can be found in Supplementary Table 1.

### Supplementary information

Supplementary Materials

## Data Availability

The data used in the manuscript can be found in Supplementary Material.
