# Supplementary material for: Disparities in clinical studies of AI enabled applications from a global perspective
Source: NPJ Digit Med. 2024 Aug 10;7:209. doi: 10.1038/s41746-024-01212-7 (PMC11316833; doi:10.1038/s41746-024-01212-7)
Supplement: Supplementary file 1 — Supplementary Materials [file 41746_2024_1212_MOESM1_ESM.pdf]

Supplementary Material

Search Strategy
("machine learning" OR "deep learning" OR "artificial intelligence" OR "data mining" OR "machine intelligence" OR "intelligent system*" OR "supervised learning" OR "natural language process*" OR "large language model*")

AND ("randomized trial*" OR "randomised trial*" OR "clinical trial*" OR "interventional trial*" OR "randomized controlled trial*" OR "randomised controlled trial*" OR "RCT" OR "RCTs" OR "implement*")

AND ("health" OR "healthcare" OR "medicine" OR "medical" OR "clinical")

Embase
Limited to "Controlled Clinical Trial" and "Randomized Controlled Trial". Limited to "Article" and "Conference Paper". Search in "All Fields". Only "English".

MEDLINE
Search in "Multi-Field Search". Limited to "Full Text". Limited to "Clinical Trial" and "Randomized Controlled Trial" related. Search in "All Fields". Only "English".

CINAHL
Limited to full-text articles. Limit to "Clinical Trial" and "Randomized Controlled Trial". Only "English".

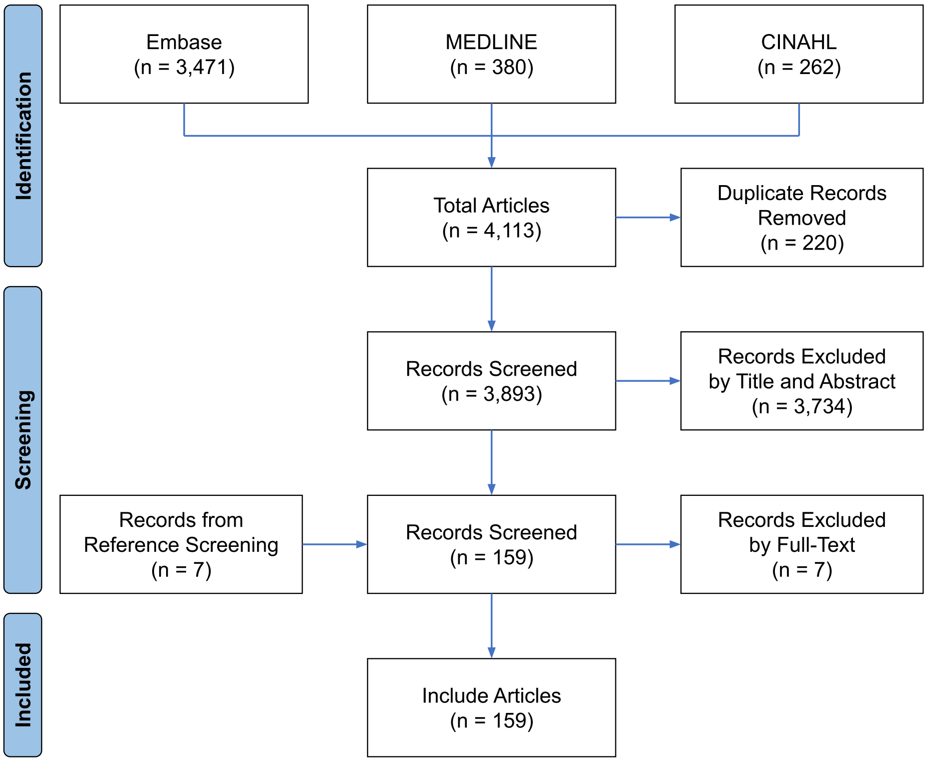

Supplementary Figure 1: PRISMA flow diagram for identification of AI-enabled clinical studies. Our search retrieved 4,113 study records (n=3,471, 84.39% from Embase; n=380, 9.24% from MEDLINE; n=234, 6.37% from CINAHL); of these, 3,893 (94.65%) were screened after deduplication (n=220, 5.35%). After the title, abstract, and full-text screening, 152 studies were retained. Furthermore, by manually reviewing the references of the included studies, another 7 related studies were identified. The total number of included studies is 159.

Supplementary Table 1: Collected information. For the collection of AI type information in studies, we only collected the specific AI algorithms that were explicitly mentioned in the studies or could be found through supplementary materials or by tracing back to previous studies. If the AI algorithm was not explicitly stated in these sources, we labeled it as "unspecified".

ID	Study	Publication Year	Collaboration	Clinical Study Country	Clinical Study – Country Income Level	Male (AI)	Female (AI)	Area of AI Application in Clinical Studies	Fund	AI Type	Training Data	Training Data - Country Income Level	Settings
1	Bai et al.	2022	No	China	Upper-middle income	18	7	Treatment and Management	Yes	VR	Unspecified		Hospital
2	Barua et al.	2022	Yes	USA	High income	88	78	Treatment and Management	Yes	Lucas-Kanade	Japan	High income	Hospital
3	Bates et al.	2023	Yes	USA	High income	12	21	Treatment and Management	Yes	Unspecified	Unspecified		Non-Hospital
4	Blomberg et al.	2021	Yes	Denmark	High income	202	116	Treatment and Management	Yes	Unspecified	Unspecified		Hospital
5	Rafael A. et al.	2018	No	Spain	High income	5	4	Treatment and Management	Yes	Unspecified	Unspecified		Hospital
6	Hernandez et al.	2020	Yes	Spain	High income	55	65	Treatment and Management	Yes	Unspecified	Unspecified		Non-Hospital
7	Chen et al.	2020	No	China	Upper-middle income	113	101	Diagnosis	Yes	CNN	China	Upper-middle income	Hospital
8	Chen et al.	2021	No	China	Upper-middle income	25	15	Diagnosis	No	CNN	Unspecified		Hospital
9	Chiang et al.	2021	No	USA	High income	16	9	Treatment and Management	Yes	Random Forest	USA	High income	Non-Hospital
10	Cho et al.	2023	Yes	South Korea	High income	N.A	N.A	Treatment and Management	Yes	XG Boost	South Korea	High income	Hospital
11	Danieli et al.	2022	No	Italy	High income	4	16	Treatment and Management	Yes	Unspecified	Unspecified		Non-Hospital
12	Backer et al.	2023	Yes	Denmark, France	High income	64	36	Treatment and Management	Yes	Unspecified	Unspecified		Hospital
13	Solh et al.	2009	No	USA	High income	25	32	Prognosis	No	ANN	USA	High income	Non-Hospital
14	Eng et al.	2021	No	USA	High income	433	359	Diagnosis	Yes	CNN	USA	High income	Hospital
15	Zaman et al.	2022	Yes	Germany	High income	215	235	Diagnosis	No	Unspecified	Unspecified		Hospital
16	Forman et al.	2019	No	USA	High income	19	100	Treatment and Management	Yes	Ensemble Learning	Unspecified		Non-Hospital
17	Forman et al.	2019	No	USA	High income	13	27	Treatment and Management	Yes	Reinforce Learning	Unspecified		Non-Hospital
18	Fuica et al.	2023	No	Israel	High income	12	24	Treatment and Management	No	Unspecified	Unspecified		Hospital
19	García et al.	2023	No	Spain	High income	82	73	Diagnosis	No	CNN	Unspecified		Hospital
20	Brown et al.	2022	Yes	USA	High income	54	59	Diagnosis	Yes	CNN	Unspecified		Hospital
21	Gong et al.	2020	No	China	Upper-middle income	187	168	Diagnosis	Yes	CNN	China	Upper-middle income	Hospital
22	Han et al.	2022	Yes	South Korea	High income	122	173	Diagnosis	No	CNN	Mainly South [illegible]	High income	Hospital
23	Hassoon et al.	2021	No	USA	High income	4	24	Treatment and Management	Yes	Unspecified	Unspecified		Non-Hospital
24	Hong et al.	2021	No	China	Upper-middle income	N.A	N.A	Treatment and Management	No	Unspecified	Unspecified		Non-Hospital
25	Hüneburg et al.	2022	No	Germany	High income	20	30	Diagnosis	Yes	Unspecified	Unspecified		Hospital
26	Hwang et al.	2023	No	South Korea	High income	978	783	Diagnosis	Yes	Unspecified	Unspecified		Hospital
27	Ishiyama et al.	2022	Yes	Japan	High income	515	403	Diagnosis	Yes	CNN	Unspecified		Hospital
28	Itoh et al.	2022	No	Japan	High income	27	21	Patient Education	Yes	Unspecified	Unspecified		Non-Hospital
29	Jaroszewski et al.	2019	No	USA	High income	N.A	N.A	Treatment and Management	Yes	RNN	Unspecified		Non-Hospital
30	Jayakumar et al.	2021	No	USA	High income	23	46	Patient Education	Yes	Unspecified	Unspecified		Hospital
31	Ji et al.	2020	No	Canada	High income	25	47	Treatment and Management	Yes	Unspecified	Unspecified		Non-Hospital
32	Kahn et al.	2022	No	USA	High income	91	42	Diagnosis	Yes	Unspecified	Unspecified		Hospital
33	Kamba et al.	2021	No	Japan	High income	272	83	Diagnosis	Yes	CNN	Unspecified		Hospital
34	Kang et al.	2023	No	South Korea	High income	22	12	Diagnosis	Yes	CNN	Unspecified		Hospital
35	Knitza et al.	2022	Yes	Germany	High income	182	418	Treatment and Management	Yes	Unspecified	Unspecified		Hospital
36	Labovtiz et al.	2017	No	USA	High income	9	6	Treatment and Management	Yes	CNN	Unspecified		Non-Hospital
37	Lee et al.	2023	No	South Korea	High income	88	74	Diagnosis	Yes	CNN	Unspecified		Hospital

38	Lee et al.	2023	No	South Korea	High income	119	62	Treatment and Management	Yes	CNN	Unspecified		Non-Hospital
39	Li et al.	2023	Yes	China	Upper-middle income	4	13	Treatment and Management	Yes	CNN; LSTM	Unspecified		Non-Hospital
40	Li et al.	2022	No	China	Upper-middle income	24	26	Treatment and Management	Yes	DNN	Unspecified		Hospital
41	Li et al.	2022	Yes	China	Upper-middle income	159	154	Treatment and Management	Yes	NLP	China	Upper-middle income	Hospital
42	Lin et al.	2022	No	China	High income	2	6	Treatment and Management	Yes	Decision Tree	Unspecified		Non-Hospital
43	Lin et al.	2019	Yes	China	Upper-middle income	77	98	Diagnosis	Yes	CNN	China	Upper-middle income	Hospital
44	Liu et al.	2022	Yes	China	Upper-middle income	19	21	Treatment and Management	No	DNN	Unspecified		Hospital
45	Liu et al.	2020	Yes	China	Upper-middle income	180	213	Diagnosis	No	CNN	Unspecified		Hospital
46	Liu et al.	2020	No	China	Upper-middle income	264	244	Diagnosis	No	CNN	China	Upper-middle income	Hospital
47	Liu et al.	2021	No	China	Upper-middle income	N.A	N.A	Treatment and Management	No	CNN	China	Upper-middle income	Hospital
48	Liu et al.	2020	No	China	Upper-middle income	197	329	Treatment and Management	Yes	Unspecified	Unspecified		Hospital
49	Lu et al.	2022	No	China	Upper-middle income	825	298	Treatment and Management	Yes	CNN	China	Upper-middle income	Hospital
50	Luo et al.	2021	No	China	Upper-middle income	76	74	Diagnosis	Yes	CNN	Unspecified		Hospital
51	Manz et al.	2020	No	USA	High income	6313	7576	Prognosis	Yes	Gradient Boosting	USA	High income	Hospital
52	Manz et al.	2023	No	USA	High income	18961	22259	Treatment and Management	Yes	Gradient Boosting	USA	High income	Hospital
53	Marcuzzi et al.	2023	No	Norway	High income	39	60	Treatment and Management	Yes	Case-based Reasoning	Unspecified		Non-Hospital
54	Medina et al.	2021	No	Spain	High income	13	2	Treatment and Management	Yes	Unspecified	Unspecified		Non-Hospital
55	Meijer et al.	2020	No	Netherlands	High income	10	15	Treatment and Management	Yes	Unspecified	Unspecified		Hospital
56	Mihailidis et al.	2008	Yes	Canada	High income	1	5	Patient Education	Yes	Unspecified	Unspecified		Non-Hospital
57	Murakami et al.	2023	No	Japan	High income	7	4	Treatment and Management	Yes	Unspecified	Unspecified		Hospital
58	Nakanuma et al.	2023	No	Japan	High income	5	5	Treatment and Management	Yes	CNN	Unspecified		Hospital
59	Nakashima et al.	2023	No	Japan	High income	153	54	Diagnosis	No	CNN	Unspecified		Hospital
60	Nakata et al.	2022	No	Japan	High income	53	19	Treatment and Management	Yes	Unspecified	Unspecified		Non-Hospital
61	Nieminen et al.	2003	No	Finland	High income	N.A	N.A	Diagnosis	Yes	DNN	Unspecified		Hospital
62	Nimri et al.	2020	Yes	USA, Slovenia, Germany, Israel	High income	28	32	Treatment and Management	Yes	Unspecified	Unspecified		Non-Hospital
63	Noseworthy et al.	2022	No	USA	High income	620	383	Diagnosis	Yes	Unspecified	Unspecified		Non-Hospital
64	Pavel et al.	2020	Yes	Ireland, Netherlands, Sweden, UK	High income	76	52	Diagnosis	Yes	Unspecified	Unspecified		Hospital
65	Pirtte et al.	2022	No	USA	High income	147	21	Treatment and Management	Yes	RL	Unspecified		Non-Hospital
66	Rafferty et al.	2021	No	USA	High income	2	12	Treatment and Management	Yes	Unspecified	Unspecified		Non-Hospital
67	Repici et al.	2020	Yes	Italy	High income	172	169	Diagnosis	No	CNN	Europe, USA	High income	Hospital
68	Repici et al.	2022	Yes	Italy, Switzerland	High income	174	156	Diagnosis	No	CNN	Europe, USA	High income	Hospital
69	Eric et al.	2021	No	USA	High income	193	60	Treatment and Management	No	Unspecified	Unspecified		Non-Hospital
70	Rondonotti et al.	2022	No	Italy	High income	213	192	Diagnosis	Yes	CNN	Unspecified		Hospital
71	Sandal et al.	2021	Yes	Denmark, Norway	High income	111	134	Treatment and Management	Yes	Case-based Reasoning	Unspecified		Non-Hospital
72	Rushlow et al.	2022	No	USA	High income	5493	6080	Diagnosis	Yes	Unspecified	Unspecified		Hospital
73	Sadasivam et al.	2016	No	USA	High income	28	46	Treatment and Management	Yes	BPMF	USA		Non-Hospital
74	Seol et al.	2021	Yes	USA	High income	53	37	Treatment and Management	Yes	NLP, Bayesian	USA	High income	Hospital
75	Shaukat et al.	2022	No	USA	High income	368	314	Diagnosis	Yes	DNN	USA	High income	Hospital
76	Shen et al.	2022	Yes	China	High income	17	16	Treatment and Management	Yes	Unspecified	Unspecified		Non-Hospital
77	Shimabukuro et al.	2017	No	USA	High income	35	32	Prognosis	Yes	Unspecified	USA	High income	Hospital

78	Singh et al.	2021	No	India	Lower-middle-income	N.A	N.A	Diagnosis	Yes	Unspecified	Unspecified		Hospital
79	Spadaccini et al.	2023	No	Italy, Switzerland	High income	298	320	Diagnosis	Yes	CNN	Europe, USA	High income	Hospital
80	Šribar et al.	2023	No	Croatia	High income	10	7	Prognosis	No	Unspecified	Unspecified		Hospital
81	Su et al.	2020	No	China	Upper-middle income	159	149	Diagnosis	Yes	CNN	China	Upper-middle income	Hospital
82	Tanaka et al.	2022	No	Japan	High income	10	22	Treatment and Management	Yes	Unspecified	Unspecified		Hospital
83	Tani et al.	2023	No	Japan	High income	306	74	Diagnosis	No	CNN	Japan	High income	Hospital
84	Tiyarattanachai et	2023	No	Thailand	Upper-middle income	146	114	Diagnosis	Yes	CNN	Thailand	Upper-middle income	Hospital
85	Turino et al.	2021	No	Spain	High income	26	4	Treatment and Management	Yes	Unspecified	Unspecified		Non-Hospital
86	Unsworth et al.	2023	No	United Kingdom	High income	21	16	Treatment and Management	Yes	Unspecified	Unspecified		Non-Hospital
87	Vilkoite et al.	2023	No	Latvia	High income	91	104	Diagnosis	Yes	CNN	Unspecified		Hospital
88	Vitral et al.	2023	Yes	Brazil, Mozambique	Upper-middle income, Low income	131	174	Diagnosis	Yes	XGBoost	Brazil	Upper-middle income	Hospital
89	Wallace et al.	2022	Yes	Italy, UK, USA	High income	157	73	Diagnosis	Yes	CNN	Unspecified		Hospital
90	Wang et al.	2019	Yes	China	Upper-middle income	263	259	Diagnosis	Yes	CNN	China	Upper-middle income	Hospital
91	Wang et al.	2020	Yes	China	Upper-middle income	179	190	Diagnosis	No	CNN	China	Upper-middle income	Hospital
92	Wang et al.	2020	Yes	China	Upper-middle income	241	243	Diagnosis	Yes	CNN	China	Upper-middle income	Hospital
93	Wang et al.	2023	No	China	Upper-middle income	364	272	Diagnosis	No	CNN	China	Upper-middle income	Hospital
94	Wang et al.	2023	No	China	Upper-middle income	N.A	N.A	Diagnosis	No	Unspecified	Unspecified		Hospital
95	Wathour et al.	2023	No	Belgium	High income	5	19	Treatment and Management	No	Unspecified	Unspecified		Hospital
96	Wijnberge et al.	2020	No	Netherlands	High income	21	10	Prognosis	Yes	Unspecified	Unspecified		Hospital
97	Wilson et al.	2023	No	USA	High income	430	325	Treatment and Management	Yes	GBM	Unspecified		Hospital
98	Wu et al.	2021	No	China	Upper-middle income	225	277	Diagnosis	Yes	CNN	China	Lower-middle-income	Hospital
99	Wu et al.	2021	Yes	China	Upper-middle income	418	489	Diagnosis	Yes	CNN	China	Upper-middle income	Hospital
100	Wu et al.	2022	No	China	Upper-middle income	28	32	Treatment and Management	Yes	CNN	Unspecified		Hospital
101	Xu et al.	2022	No	China	Upper-middle income	35	41	Treatment and Management	Yes	CNN	China	Upper-middle income	Hospital
102	Xu et al.	2023	Yes	China	Upper-middle income	707	812	Diagnosis	No	CNN	China	Upper-middle income	Hospital
103	Xu et al.	2021	No	China	Upper-middle income	603	574	Diagnosis	Yes	CNN	Unspecified		Hospital
104	Xu et al.	2022	No	China	Upper-middle income	24	19	Treatment and Management	No	Unspecified	Unspecified		Hospital
105	Yacoub et al.	2022	Yes	USA	High income	102	93	Diagnosis	Yes	CNN	Unspecified		Hospital
106	Yang et al.	2022	No	China	Upper-middle income	464	498	Diagnosis	Yes	CNN	Unspecified		Hospital
107	Yao et al.	2021	No	China	Upper-middle income	378	427	Diagnosis	Yes	CNN	China	Upper-middle income	Hospital
108	Yao et al.	2021	Yes	USA	High income	5493	6080	Diagnosis	Yes	CNN	Unspecified		Hospital
109	Yoon et al.	2022	No	South Korea	High income	11	22	Patient Education	Yes	CNN	Unspecified		Non-Hospital
110	Zhang et al.	2022	No	China	Upper-middle income	17	9	Diagnosis	Yes	CNN	Unspecified		Hospital
111	Wu et al.	2023	No	China	Upper-middle income	81	63	Diagnosis	Yes	CNN	China	Upper-middle income	Hospital
112	Zhu et al.	2023	No	China	Upper-middle income	126	127	Treatment and Management	Yes	CNN	China	Upper-middle income	Non-Hospital
113	Zhu et al.	2022	No	China	Upper-middle income	N.A	N.A	Treatment and Management	No	CNN	Unspecified		Hospital
114	Avari et al.	2021	Yes	UK, Spain	High income	24	26	Treatment and Management	Yes	Unspecified	Unspecified		Non-Hospital
115	Strömblad et al.	2021	No	USA	High income	54	273	Treatment and Management	Yes	Random Forest	USA	High income	Hospital
116	Alfonsi et al.	2020	No	Canada	High income	11	11	Treatment and Management	Yes	CNN	Unspecified		Non-Hospital

117	Anan et al.	2021	No	Japan	High income	39	9	Treatment and Management	Yes	Unspecified	Unspecified		Non-Hospital
118	Auloge et al.	2020	Yes	France	High income	3	7	Treatment and Management	Yes	Unspecified	Unspecified		Hospital
119	Benhamou et al.	2019	No	France	High income	24	39	Treatment and Management	Yes	Unspecified	Unspecified		Non-Hospital
120	Faro et al.	2023	No	USA	High income	201	540	Treatment and Management	Yes	Unspecified	Unspecified		Non-Hospital
121	Horne et al.	2022	No	USA	High income	61	28	Treatment and Management	Yes	Reinforce Learning	Unspecified		Non-Hospital
122	Chan et al.	2021	No	Singapore	High income	N.A	N.A	Treatment and Management	Yes	Unspecified	Unspecified		Hospital
123	Kränke et al.	2023	No	Austria	High income	152	86	Diagnosis	Yes	CNN	Unspecified		Hospital
124	Li et al.	2022	No	China	Upper-middle income	8813	8974	Diagnosis	Yes	CNN	China	Upper-middle income	Hospital
125	Liu et al.	2022	No	China	Upper-middle income	17	24	Treatment and Management	No	NLP	Unspecified		Non-Hospital
126	Martin et al.	2012	Yes	Ireland	High income	49	104	Treatment and Management	No	Unspecified	Unspecified		Hospital
127	Gandía et al.	2018	No	Spain	High income	6	6	Treatment and Management	Yes	DNN	Spain	High income	Non-Hospital
128	Remmers et al.	2017	No	Canada	High income	40	8	Diagnosis	Yes	Random Forest	Canada	High income	Non-Hospital
129	Wu et al.	2019	No	China	Upper-middle income	76	77	Treatment and Management	Yes	CNN	China	Upper-middle income	Hospital
130	Vennalaganti et al.	2018	No	USA	High income	122	38	Diagnosis	Yes	DNN	Unspecified		Hospital
131	Persell et al.	2020	Yes	USA	High income	53	91	Treatment and Management	Yes	Unspecified	Unspecified		Non-Hospital
132	Voss et al.	2019	No	USA	High income	37	3	Treatment and Management	Yes	Unspecified	Unspecified		Non-Hospital
133	Nicolae et al.	2020	No	Canada	High income	N.A	N.A	Treatment and Management	Yes	Unspecified	Unspecified		Hospital
134	Browning et al.	2021	Yes	UK, Spain, Germany, France, Netherlands	High income	175	285	Treatment and Management	Yes	SVM	UK, Spain, Germany, France, Netherlands	High income	Non-Hospital
135	Kamba et al.	2021	No	Japan	High income	272	83	Diagnosis	Yes	CNN	Japan	High income	Hospital
136	Granviken et al.	2023	No	Norway	High income	3	9	Treatment and Management	Yes	Unspecified	Unspecified		Non-Hospital
137	Joshi et al.	2023	Yes	India	Lower-middle-income	195	38	Treatment and Management	Yes	Gradient Boost, DNN	Unspecified		Non-Hospital
138	Nayak et al.	2023	No	USA	High income	7	9	Treatment and Management	No	Unspecified	Unspecified		Non-Hospital
139	Gutierrez et al.	2023	No	USA	High income	121	122	Diagnosis	Yes	Unspecified	Unspecified		Hospital
140	Huang et al.	2023	No	China	Upper-middle income	5374	4521	Treatment and Management	Yes	Unspecified	Unspecified		Hospital
141	Sanjuan et al.	2023	No	Spain	High income	865	745	Diagnosis	Yes	CNN	Unspecified		Hospital
142	Sharma et al.	2023	No	Canada	High income	36	16	Treatment and Management	Yes	Unspecified	Unspecified		Hospital
143	Wei et al.	2023	Yes	USA	High income	194	193	Diagnosis	No	CNN	Unspecified		Hospital
144	Yang et al.	2023	Yes	China	Upper-middle income	398	210	Treatment and Management	Yes	Unspecified	Unspecified		Hospital
145	Hilli et al.	2023	No	USA	High income	N.A	N.A	Treatment and Management	Yes	Unspecified	Unspecified		Non-Hospital
146	Lång et al.	2023	Yes	Sweden	High income	N.A	N.A	Diagnosis	Yes	Unspecified	Unspecified		Hospital
147	Sharvit et al.	2023	No	USA	High income	7	16	Treatment and Management	No	Unspecified	Unspecified		Non-Hospital
148	Karsenti et al.	2023	No	France	High income	481	522	Diagnosis	No	Unspecified	Unspecified		Hospital
149	Sandhu et al.	2023	No	USA	High income	42	44	Diagnosis	Yes	Unspecified	Unspecified		Hospital
150	Cho et al.	2023	No	South Korea	High income	26980	28103	Prognosis	Yes	Unspecified	unspecified		Hospital
151	Hsu et al.	2023	No	China	High income	12	6	Prognosis	Yes	MLP	China	Upper-middle income	Hospital
152	Yamaguchi et al.	2023	No	Japan	High income	58	55	Diagnosis	Yes	CNN	Unspecified		Hospital
153	Nam et al.	2023	No	South Korea	High income	2592	2646	Diagnosis	Yes	Unspecified	Unspecified		Hospital
154	Hong et al.	2020	No	USA	High income	89	65	Treatment and Management	Yes	Gradient Boost	USA	High income	Hospital
155	Luna et al.	2021	Yes	USA	High income	8	7	Treatment and Management	Yes	Unspecified	Unspecified		Non-Hospital

156	Wang et al.	2019	No	USA	High income	N.A	N.A	Treatment and Management	Yes	Unspecified	Unspecified		Hospital
157	Ahmad et al.	2023	No	UK	High income	110	198	Diagnosis	Yes	Unspecified	Unspecified		Hospital
158	Abramoff et al.	2023	Yes	Bangladesh	Lower-middle-income	235	259	Treatment and Management	Yes	Unspecified	Unspecified		Hospital
159	Tsoumpa et al.	2021	No	Greece	High income	26	23	Prognosis	No	Unspecified	Unspecified		Hospital

Supplementary Table 2: Related disease in studies with high gender disparity.

Male Majority		Female majority	
Medina et al.	Attention Deficit Hyperactivity Disorder	Danieli et al.	Stress and Anxiety
Pirtte et al.	Chronic Pain	Forman et al.	Obesity
Tani et al.	Esophageal Squamous Cell Carcinoma	Hassoon et al.	Obesity in Cancer Survivors
Turino et al.	Obstructive Sleep Apnea	Mihailidis et al.	Dementia
Anan et al.	Neck/Shoulder Pain/Stiffness and Low Back Pain	Rafferty et al.	Irritable Bowel Syndrome
Remmers et al.	Obstructive Sleep Apnea	Wathour et al.	Post-lingual Deafness
Voss et al.	Autism Spectrum Disorder	Strömblad et al.	Colorectal and Gynecology Surgery
Joshi et al.	Type 2 Diabetes		
